# Integrated glycomic analysis of ovarian cancer side population cells

**DOI:** 10.1186/s12014-016-9131-z

**Published:** 2016-11-10

**Authors:** Ran Zhao, Xiaoxia Liu, Yisheng Wang, Xiaoxiang Jie, Ruihuan Qin, Wenjun Qin, Mengyu Zhang, Haiyan Tai, Caiting Yang, Lili Li, Peike Peng, Miaomiao Shao, Xingwang Zhang, Hao Wu, Yuanyuan Ruan, Congjian Xu, Shifang Ren, Jianxin Gu

**Affiliations:** 1 Institute of Biomedical Sciences, Fudan University, 138 Yi-Xueyuan Road, Shanghai, 200032 People’s Republic of China; 2 Obstetrics and Gynecology Hospital of Fudan University, 419 Fang-Xie Road, Shanghai, 200011 People’s Republic of China; 3 Key Laboratory of Glycoconjugate Research Ministry of Public Health, Department of Biochemistry and Molecular Biology, School of Basic Medical Sciences, Fudan University, 138 Yi-Xueyuan Road, Shanghai, 200032 People’s Republic of China; 4 Department of Obstetrics and Gynecology of Shanghai Medical School, Fudan University, Shanghai, 200032 People’s Republic of China; 5 Shanghai Key Laboratory of Female Reproductive Endocrine Related Diseases, Shanghai, 200032 People’s Republic of China

**Keywords:** Ovarian cancer stem cells, Glycomic, Biomarker, Tn antigen, T antigen, sT antigen

## Abstract

**Background:**

Ovarian cancer is the most lethal gynecological malignancy due to its frequent recurrence and drug resistance even after successful initial treatment. Accumulating scientific evidence indicates that subpopulations of cancer cells with stem cell-like properties, such as so-called side population (SP) cells, are primarily responsible for these recurrences. A better understanding of SP cells may provide new clues for detecting and targeting these cancer-initiating cells and ultimately help to eradicate cancer. Changes in glycosylation patterns are remarkable features of SP cells. Here, we isolated SP cells from ovarian cancer cell lines and analyzed their glycosylation patterns using multiple glycomic strategies.

**Methods:**

Six high-grade serous ovarian cancer cell lines were used for SP cell isolation. Among them, HO8910 pm, which contained the highest proportion of SP cells, was used for glycomic analysis of SP cells. Cell lysate of SP cells and main population cells was applied to lectin microarray and mass spectrometry for glycan profiling. Differently expressed glycan structures were further verified by lectin blot, flow cytometry, and real-time PCR analysis of their relevant enzymes.

**Results:**

Expression of core fucosylated N-glycan and tumor-associated Tn, T and sT antigens were increased in SP cells. By contrast, SP cells exhibited decreased hybrid glycan, α2,3-linked sialic glycan and multivalent sialyl-glycan.

**Conclusions:**

Glycan structures, such as Tn, T, sT antigens, and core fucosylation may serve as biomarkers of ovarian cancer stem cells.

**Electronic supplementary material:**

The online version of this article (doi:10.1186/s12014-016-9131-z) contains supplementary material, which is available to authorized users.

## Background

Ovarian cancer is one of the most lethal gynecological malignancies [1]. The high mortality rate of ovarian cancer has been attributed both to late diagnosis due to lack of early symptoms and to recurrence accompanied by drug resistance [2–4]. Although 80% of patients are initially responsive to optimal cytoreductive surgery plus platinum-based chemotherapy, recurrent cancer is inevitable in vast majority of cases. Along with recurrence, drug resistance develops, leading to death in 70% of ovarian cancer patients [5]. Thus, recurrence and drug resistance are main challenges in ovarian cancer treatment.

Accumulating scientific evidence suggests that tumors are driven by relatively rare and biologically distinct cancer-initiating cells, so-called cancer stem cells (CSCs), which are capable of self-renewal and further differentiation [6–8]. These cells are resistant to regular chemotherapy because they remain quiescent for a long time, proliferate slowly and excrete drugs easily [7, 8]. Detecting and eliminating CSCs would prohibit relapse and improve survival of ovarian cancer patients [9].

Glycosylation is the most prevalent post-translational modification involved in many physiological and pathological processes. A large number of studies have demonstrated that glycans participate in cancer initiation [10], development [11–14], and drug resistance [15]. In addition, several reports have suggested that glycans and glycosyltransferases play important roles in CSC function. For example, increased fucosylation promotes invasive and metastatic properties of head and neck cancer stem cells [16]. β1,4-*N*-Acetylgalactosaminyltransferase (GalNAcT) III modulates the stemness of colon cancer via EGFR signaling pathway [17]. Glycans can also serve as potential CSC biomarkers. In combination with traditional biomarkers, glycans can be used to isolate more malignant CSCs. For example, anti-CD133 antibody, together with SSA (*Sambucus sieboldiana*) lectin, can identify CSCs of higher tumourigenicity in hepatocellular carcinoma [18]. Moreover, expression pattern of certain glycans on CSCs is distinct from that on normal stem cells, thus may improve the specificity of detecting and targeting CSCs [19]. Consequently, glycan biomarkers have more advantages with fewer side effects to harm the normal stem cells than general CSC markers. Glycomic analysis may provide comprehensive glycome profiles and potential biomarkers of CSCs. The specific glycans of CSCs have potential clinical value as targets in patients undergoing chemotherapy.

So far, little is known about glycosylation of ovarian CSCs. In this study, an integrated strategy using lectin array and mass spectrometry (MS) was employed to obtain the global glyco-information of ovarian cancer side population (SP) cells. Core fucosylated N-glycan and tumor-associated Tn, T and sT antigens were increased, while hybrid glycan, α2,3-linked sialic glycan and multivalent sialyl-glycan were decreased in SP cells. These differentially expressed glycans may serve as potential biomarkers of ovarian cancer SP cells.

## Methods

### Cell culture

The human high-grade serous ovarian cancer cell lines SKOV3 and HO8910 and their highly metastatic sublines SKOV3 ip and HO8910 pm were purchased from the cell bank of Shanghai Institute of Biochemistry and Cell Biology (China). The high-grade ovarian cancer cell line A2780 and its cisplatin-resistant subline A2780-cp were obtained from Obstetrics and Gynecology Hospital of Fudan University (China). All cell lines were routinely cultured in DMEM (Gibco Life Technologies, Carlsbad, CA, USA) supplemented with 10% foetal bovine serum (Gibco) and 100 U/ml penicillin/streptomycin (Gibco).

### Side population sorting

SP sorting was performed as previously described [20]. Briefly, the fluorescent dye Hoechst 33342 (Sigma-Aldrich, St. Louis, MO, USA) was added to a cell suspension (1 × 10^6^ cells/ml) at a final concentration of 5 μg/ml. After incubation at 37 °C for 90 min, the cells were treated with 2 μg/ml propidium iodide (PI) and sorted on a MoFlo XDP cytometer (Beckman Coulter, Brea, CA, USA). Cells treated with verapamil before Hoechst 33342 staining were used as negative control. Main population cells at the G0 stage were collected.

### Spheroid formation assay

SP cells or MP cells were seeded at 2000 per well in 24-well Ultra Low plates (Corning, New York, NY, USA) in DMEM/F12 medium (Gibco) containing 20 ng/ml fibroblast growth factor, 20 ng/ml epidermal growth factor, 2% B27, insulin (R&D Systems, Minneapolis, MN, USA) and 2 mg/ml heparin (Sigma-Aldrich) and cultured for 21 days.

### Cell cycle analysis

After cell sorting, 5 × 10^5^ SP cells and MP cells were suspended in phosphate-buffered saline (PBS) buffer containing 50 μg/ml PI dividedly, incubated in the dark for 30 min at room temperature, and analyzed with a CyAn ADP flow cytometer (Beckman Coulter). Data were analyzed with ModFit LT for Windows version 3.1.

### Lectin microarray

After sorting, SP cells and MP cells were washed several times with PBS and frozen at −80 °C. For lectin microarray analysis, 1 × 10^5^ cells were resuspended in 1 ml of PBS-T (containing 1% TritonX-100), followed by sonication for 15 min and centrifugation at 14,000*g* for 15 min. The supernatant was recovered. Proteins were quantitated using a micro BCA kit (Thermo Scientific) and labelled with fluorescent dye Cy3 (Thermo Scientific). Samples with protein concentration of 250 ng/ml were applied to a LecChip (Glyco Technica) and incubated at 20 °C for 16 h. The chip was then scanned with a GlycoStation Reader 1200 (Glyco Technica) confocal scanner. Each lectin in LecChip has three replicates. To be normalized, intensity of each well in lectin microarray was divided by the mean of total 135 wells’ intensity of the chip. We repeated lectin microarray analysis of SP and MP cells using independent samples to overcome biological bias.

### Cell lysis preparation for mass spectrometry analysis

SP cells were rinsed with PBS. After washing, 2% SDS containing protease inhibitor cocktail (Roche Diagnostics, Roche Applied Science, Meylan, France) was used to lyse the cells at 100 °C for 15 min. The lysate was then centrifuged at 14,000*g* for 30 min, and the supernatant was collected. The protein concentration in the supernatant was quantitated using a BCA kit (Thermo Scientific, San Jose, CA, USA).

### N-Glycan release and purification

For each cell line, 400 μg of protein in 200 μl of 2% SDS was added to 200 μl of 8 M urea (Sigma-Aldrich) containing dithiothreitol (Sigma-Aldrich) to achieve a final concentration of 10 mM. After heating at 56 °C for 20 min, the samples were incubated in 40 mM ammonium bicarbonate (Sigma-Aldrich) containing 25 mM iodoacetamide (Sigma-Aldrich) for 30 min at 37 °C in the dark. The sample was transferred to an ultrafiltration unit (Amicon Ultra-0.5, Ultracel-10 membrane; Millipore, Billerica, MA) and centrifuged at 14,000*g* for 15 min. A volume of 200 μl of 40 mM NH_4_HCO_3_ was added to the ultrafiltration unit and centrifuged to wash the sample. Thereafter, 2 μl of PNGase F (New England BioLab, Ipswich, MA) in 200 μl of 40 mM NH_4_HCO_3_ was added to the device and incubated with shaking for 24 h at 37 °C. The ultrafiltration unit was transferred to a new collection tube and centrifuged at 14,000*g* for 15 min, and the filter membrane was washed with 200 μl of 40 mM NH_4_HCO_3_ for three times. The solution in the collection tube was recovered and lyophilizedin a vacuum freeze dryer (Martin Christ GmbH, Osterode, Germany). To remove sialic acids, each sample was reconstituted in 50 mM ammonium acetate buffer (pH 5.5) followed by desialylation with neuraminidase (15 mU) from *V. cholerae* (Roche) (Sigma-Aldrich, St. Louis, MO) at 37 °C overnight. Subsequently, all samples were dried in a SpeedVac and redissolved in 50 μl of water (with 0.1% TFA).

The N-glycans in solution were purified and desalted using a Porous Graphic Carbon Solid-Phase Extraction (PGC-SPE) as previously described [21]. The PGC-SPE microcolumn was a GELoader tip filled with porous graphic carbon powder. The microcolumn was prepared with 6 volumes of 0.1% (v/v) trifluoroacetic acid (TFA) in 80% acetonitrile (ACN)/H_2_O (v/v) and equilibrated with water. The N-glycan solution was passed through the microcolumn 5 times to ensure complete adsorption. The N-glycans were eluted with 400 μl of 0.05% (v/v) TFA in 25% (v/v) ACN and lyophilized.

### Mass spectrometry analysis

N-Glycans were characterized by AXIMA Resonance MALDI QIT TOF MS (Shimadzu Corp, Kyoto, Japan). The lyophilized desialylated glycans were resuspended in 20 μl of water. Each sample (1 μl) was spotted on a MALDI target and dried in air at room temperature. To recrystallize the glycans, 1 μl of 12.5 mg/ml 2,5-dihydroxybenzoic acid matrix (DHB, Sigma-Aldrich, Germany) dissolved in 50% ACN containing 0.1% TFA was added to the sample target. Each sample was spotted in five replicates. Glycan structures were analyzed using Glyco Workbench (http://code.google.com/p/glycoworkbench/).

Launchpad software (Shimadzu Biotech, Kyoto, Japan) was used to acquire and process the MALDI MS data. The relative peak intensities were calculated as previously described [22, 23], that relative intensity of each type of N-glycan was calculated by dividing the intensity of a given type of N-glycan by sum of the total 25 N-glycans intensity. Relative standard deviation (RSD) percentages based on relative intensity values were used to estimate the stability of mass spectrometry. Relative intensity of the five replicates of a certain N-glycan were averaged and compared between SP and MP cells. *p* < 0.05 were considered statistically significant. SP/MP ratios were calculated to express magnitude of changes. Cut-off of significant changes was set as >1.5 or <0.67 folds.

### Lectin blot

The proteins isolated from SP cells and MP cells were analyzed by SDS-PAGE and lectin blot. In brief, samples were mixed with 5× loading buffer, boiled and separated by 10% SDS–polyacrylamide gel electrophoresis. The proteins in the gels were transferred to PVDF membranes (Millipore, Bedford, MA, USA). The PVDF membranes were blocked with TBST (150 mM NaCl, 10 mM Tris–HCl, 0.05% v/v Tween 20, pH 7.5) containing 5% bovine serum albumin for 1 h at room temperature and incubated with biotinylated *Agaricus bisporus* (ABA; Vector Laboratories, Burlingame, CA) or *Vicia villosa* lectin (VVA; Vector Laboratories) for 2 h at room temperature. After washing with TBST for 3 times, the membranes were incubated with horseradish peroxidase streptavidin (Vector Laboratories) for 30 min at room temperature. Signals were subsequently detected using an ECL assay kit.

### Total RNA extraction and quantitative real-time PCR analysis

Total RNA was extracted with TRIzol™ reagent (Invitrogen, Carlsbad, CA, USA), and 2 μg of RNA was used to synthesize cDNA using an RT Master Mix kit (Takara, Shiga, Japan). Real-time PCR analysis was performed using an ABI 7500 Fast Real-time PCR system (Applied Biosystems, Switzerland) for 40 cycles (15 s at 95 °C, 30 s at 60 °C). A 2-μl aliquot of cDNA was amplified in 20 μl system using the SYBR-green Premix Real-time PCR kit (Takara) system according to the manufacturer’s instruction. The primer sequences were as follows: GAPDH, GTCAAGGCTGAGAACGGGAA (forward) and AAATGAGCCCCAGCCTTCTC (reverse); Oct4, GACAACAATGAAAATCTTCAGGAGA (forward) and TTCTGGCGCCGGTTACAGAACCA (reverse); Nanog, CAAAGGCAAACAACCCACTT (forward) and TCTGCTGGAGGCTGAGGTAT (reverse); Fut8, TCTTCATCCCCGTCCTCCA (forward) and GAGACACCCACCACACTGCA (reverse); C1galt1, CATCCCTTTGTGCCAGAACACC (forward) and GCAAGATCAGAGCAGCAACCAG (reverse); St3gal1, GGGCAGACAGCAAAGGGAA (forward) and GGCCGTCACGTTAGACTCAAA (reverse).

### Flow cytometry

After sorting, the SP cells and MP cells were cultured for 5 weeks in the previously mentioned medium in 6-well Ultra Low plates (Corning). The spheres were divided into single cells, stained with fluorescent-labelled ABA and VVA (Vector Laboratories) for 15 min at room temperature, and detected using CyAn ADP (Beckman Coulter).

### Statistical analyses

Means of continuous data were compared using Student’s *t* test by SPSS software (version 16.0), *p* < 0.05 was considered statistically significant. The results shown in figures were expressed as mean ± SD.

## Results

### Ovarian cancer SP cells exhibit stem cell-like properties

**Fig. 1:**
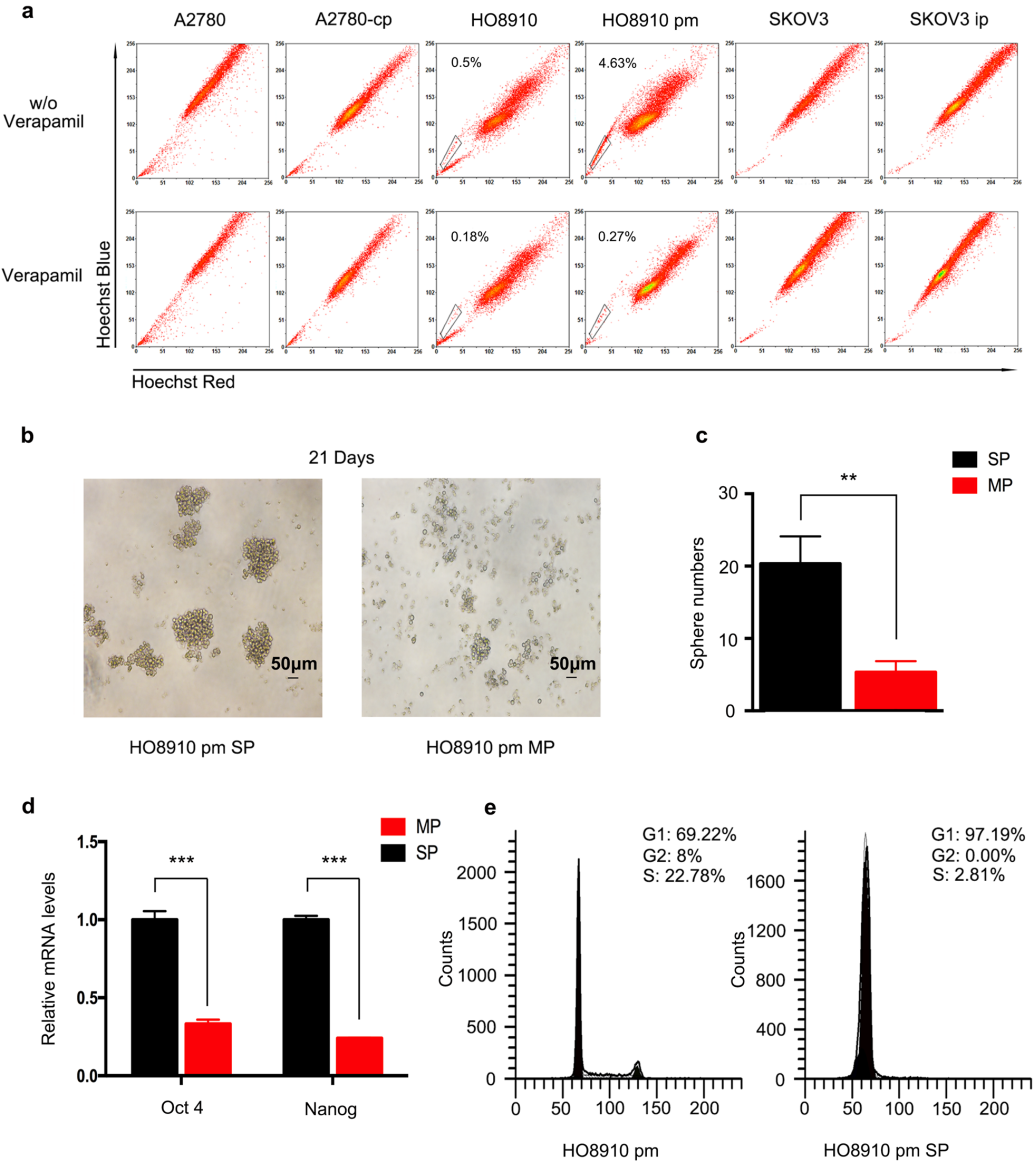
Separation of SP cells from the HO8910 pm cell line and verification of their stemness. **a** Detection of SP cells from high-grade serous ovarian cancer cells lines. Cells were stained with the dye Hoechst 33342 and analyzed by flow cytometry. The ratio of SP cells was highest in HO8910 pm cells. **b** The spheres that formed after 21 days of culturing in an ultra-low attachment plate were much larger of SP cells than MP cells. **c** More spheres formed of SP cells than MP cells. **d** The mRNA expression of Oct4 and Nanog was up-regulated in SP cells. **e** SP cells were arrested in G1 stage

To enrich SP cells, we analyzed six serous ovarian cancer cell lines, including SKOV3, HO8910 and their highly metastatic derivatives SKOV3 ip and HO8910 pm, as well as A2780 and its drug-resistant derivative A2780 cp. HO8910 pm cells exhibited 4.63% Hoechst low staining SP cells, as previously reported [20] (Fig. 1a). Other cell lines exhibited only small amounts of SP cells, which were inadequate for glycoprofiling. Thus, we chose HO8910 pm cells for further studies.

Compared with the main population (MP) cells, SP cells exhibited higher self-renewal capacity. After plating in ultra-low attachment plates for 3 weeks, the SP cells formed significantly larger numbers of spheroids than MP cells. Moreover, the spheroids of SP cells were much larger in size (Fig. 1b, c). To further characterize the stem cell-like features of SP cells, we analyzed the expression of stem genes using real-time PCR. Both Oct-4 and Nanog were over-expressed in SP cells (Fig. 1d). CSCs are considered to reside in a stable quiescent state in the G0 state of the cell cycle and are therefore resistant to chemotherapies [7]. We also examined the cell cycle of SP cells: nearly 97.5% were arrested in the G0/G1 state (Fig. 1e), whereas only 66.17% of unsorted HO8910 pm cells were in the G1 state.

### Lectin array analysis of SP cells and MP cells

**Fig. 2:**
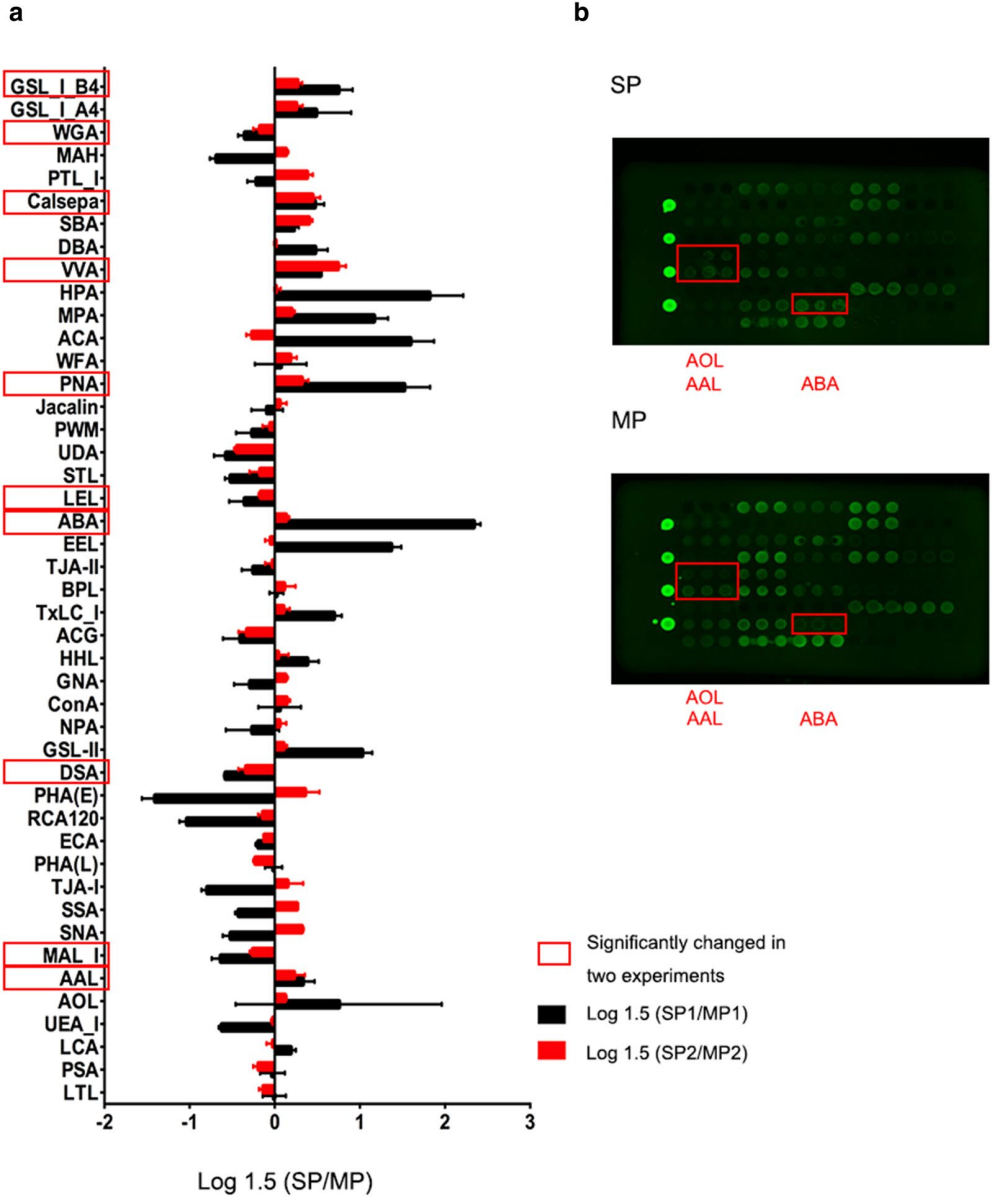
Lectin array analysis of SP cells and MP cells. **a** Relative intensities of 45 lectins in SP cells compared with MP cells for two experiments. **b** Fluorescence intensities of SP cells (*up*) and MP cells (*down*)

To obtain more comprehensive information about the global glycosylation of SP cells, lectin array including 45 lectins were analyzed. Signal intensity of 9 lectins were significantly different between SP and MP cells in both of the two experiments (Fig. 2; Table 1).

**Table 1:** Variation of glycans determined by lectin microarray analysis of SP cells and MP cells

No.	Lectin	Preferred glycan structure (terminal epitope)	SP1 ± SD	MP1 ± SD	SP1/MP1	SP2 ± SD	MP2 ± SD	SP2/MP2
1	LTL	Fuc α1-3(Gal β1-4)GlcNAc (Le^x^), Fuc α1-2(Gal β1-4)GlcNAc	0.40 ± 0.02	0.40 ± 0.02	1.00	0.42 ± 0.02	0.45 ± 0.02	0.95
2	PSA	Fuc α1-6GlcNAc, α-D-Glc, α-D-Man	0.78 ± 0.01	0.79 ± 0.06	0.99	0.82 ± 0.04	0.88 ± 0.02	0.93
3	LCA	Fuc α1-6GlcNAc, α-D-Glc, α-D-Man	0.91 ± 0.07	0.84 ± 0.06	1.08	0.89 ± 0.01	0.89 ± 0.03	0.99
4	UEA-I	Fuc α1-2(Gal β1-4)GlcNAc	0.41 ± 0.02	0.53 ± 0.03	0.78**	0.60 ± 0.02	0.61 ± 0.01	0.99
5	AOL	Fuc α1-6GlcNAc (core Fuc), Fuc α1-2(Gal β1-4)GlcNAc	0.93 ± 0.34	0.65 ± 0.04	1.43	0.58 ± 0.04	0.55 ± 0.04	1.05
6	AALª	Fuc α1-6GlcNAc (core Fuc), Fuc α1-3(Gal β1-4)GlcNAc (Le^x^)	1.22 ± 0.09	1.07 ± 0.04	1.14*	0.95 ± 0.05	0.87 ± 0.01	1.10*
7	MALª	Sia α2-3Gal β1-4GlcNAc	0.47 ± 0.02	0.60 ± 0.01	0.77***	0.63 ± 0.02	0.70 ± 0.01	0.90**
8	SNA	Sia α2-6Gal/GalNAc	0.82 ± 0.02	1.01 ± 0.05	0.81**	1.03 ± 0.03	0.90 ± 0.03	1.14**
9	SSA	Sia α2-6Gal/GalNAc	0.75 ± 0.04	0.90 ± 0.06	0.84*	1.03 ± 0.01	0.92 ± 0.01	1.11***
10	TJA-I	Sia α2-6Gal/GalNAc	1.65 ± 0.01	2.28 ± 0.08	0.73***	1.66 ± 0.15	1.56 ± 0.06	1.06
11	PHA-L	Tri/Tetra-antennary complex-type N-glycan	0.45 ± 0.03	0.45 ± 0.01	1.00	0.54 ± 0.01	0.59 ± 0.01	0.91**
12	ECA	Gal β1-4GlcNAc	0.86 ± 0.04	0.93 ± 0.04	0.93	1.12 ± 0.04	1.18 ± 0.04	0.95
13	RCA120	Gal β1-4GlcNAc	1.60 ± 0.07	2.43 ± 0.20	0.66**	2.13 ± 0.08	2.25 ± 0.12	0.95
14	PHA-E	Complex-type N-glycans with outer Gal and bisecting GlcNAc	0.75 ± 0.05	1.33 ± 0.06	0.57***	1.01 ± 0.02	0.88 ± 0.05	1.15*
15	DSAª	(GlcNAc β1-4)_n_, Gal β1-4GlcNAc, Tri/Tetra-antennary N-glycans	1.75 ± 0.03	2.21 ± 0.06	0.79***	1.78 ± 0.04	2.04 ± 0.04	0.87**
16	GSL-II	Agalactosylated tri/tetra antennary glycans, GlcNAc	0.48 ± 0.02	0.31 ± 0.00	1.52***	0.44 ± 0.02	0.43 ± 0.01	1.04
17	NPA	High Man, Man α1-6Man	1.52 ± 0.17	1.68 ± 0.03	0.90	1.41 ± 0.04	1.38 ± 0.07	1.02
18	ConA	High Man, Man α1-6(Man α1-3)Man (inhibited by presence of bisecting GlcNAc)	2.29 ± 0.23	2.23 ± 0.16	1.03	1.68 ± 0.11	1.59 ± 0.09	1.06
19	GNA	High Man, Man α1-3Man	0.98 ± 0.08	1.10 ± 0.01	0.89	1.02 ± 0.02	0.97 ± 0.03	1.05
20	HHL	High Man, Man α1-3Man, Man α1-6Man	0.67 ± 0.04	0.58 ± 0.01	1.17*	0.75 ± 0.02	0.74 ± 0.03	1.01
21	ACG	Sia α2-3Gal β1-4GlcNAc	1.36 ± 0.18	1.59 ± 0.09	0.85	1.38 ± 0.07	1.57 ± 0.05	0.88*
22	TxLC-I	Man_3_ core, bi- and tri-antennary complex-type N-glycan, GalNAc	1.06 ± 0.04	0.80 ± 0.05	1.32**	0.97 ± 0.05	0.93 ± 0.04	1.04
23	BPL	Gal β1-3GalNAc (α-Thr/Ser (T)), GalNAc	0.54 ± 0.05	0.53 ± 0.03	1.01	0.66 ± 0.02	0.63 ± 0.02	1.05
24	TJA-II	Fuc α1-2Gal β1-4, GalNAcβ1-4 groups at their nonreducing terminals	0.92 ± 0.04	1.01 ± 0.02	0.90*	1.11 ± 0.06	1.12 ± 0.03	0.99
25	EEL	Gal α1-3Gal β1-4GlcNAc, Fuc α1-2(Gal α1-3)Galβ1-4GlcNAc	0.58 ± 0.04	0.33 ± 0.02	1.74**	0.51 ± 0.02	0.52 ± 0.01	0.98
26	ABAª	Gal β1-3GalNAc (α-Thr/Ser (T)), GlcNAc, sialyl-T	2.16 ± 0.08	0.84 ± 0.06	2.58***	0.89 ± 0.01	0.84 ± 0.01	1.06*
27	LELª	(GlcNAc β1-4)_n_, (Gal β1-4GlcNAc)_n_ (polyLacNAc)	2.39 ± 0.12	2.75 ± 0.16	0.87*	2.95 ± 0.08	3.16 ± 0.09	0.94*
28	STL	(GlcNAc)_n_, (GlcNAc β1-4MurNAc)_n_ (peptidoglycan backbone)	1.77 ± 0.13	2.19 ± 0.14	0.81*	2.21 ± 0.16	2.36 ± 0.09	0.94
29	UDA	GlcNAc β1-4GlcNAc, Mixture of Man_5_ to Man_9_	1.65 ± 0.16	2.09 ± 0.32	0.79	1.62 ± 0.21	1.94 ± 0.26	0.84
30	PWM	(GlcNAc α1-4)_n_	0.52 ± 0.04	0.58 ± 0.08	0.90	0.82 ± 0.00	0.84 ± 0.03	0.98
31	Jacalin	Gal β1-3GalNAc (α-Thr/Ser (T)), GalNAc (α-Thr/Ser (Tn))	1.51 ± 0.25	1.55 ± 0.15	0.97	1.64 ± 0.04	1.60 ± 0.09	1.02
32	PNAª	Gal β1-3GalNAc (α-Thr/Ser (T))	0.68 ± 0.09	0.36 ± 0.01	1.86**	0.49 ± 0.01	0.43 ± 0.01	1.14**
33	WFA	Terminal GalNAc (e.g. GalNAcβ1-4GlcNAc), Galβ1-3(-6)GalNAc	0.52 ± 0.05	0.51 ± 0.02	1.03	0.71 ± 0.08	0.66 ± 0.06	1.08
34	ACA	Gal β1-3GalNAc (α-Thr/Ser (T))	2.20 ± 0.15	1.16 ± 0.14	1.90***	0.87 ± 0.07	0.97 ± 0.05	0.90
35	MPA	Gal β1-3GalNAc (α-Thr/Ser (T)), GalNAc (α-Thr/Ser (Tn))	0.81 ± 0.08	0.50 ± 0.02	1.61**	0.71 ± 0.04	0.66 ± 0.04	1.08
36	HPA	α-Linked terminal GalNAc	0.89 ± 0.04	0.43 ± 0.05	2.08***	0.45 ± 0.03	0.45 ± 0.02	1.01
37	VVAª	α-linked terminal GalNAc (α-Thr/Ser (Tn), GalNAc α1-3Gal	0.55 ± 0.03	0.44 ± 0.02	1.24**	0.59 ± 0.03	0.43 ± 0.00	1.35***
38	DBA	GalNAc α1-3GalNAc (Blood group A), GalNAc α1-3GalNAc	0.41 ± 0.04	0.34 ± 0.01	1.21*	0.46 ± 0.01	0.46 ± 0.01	1.00
39	SBA	α or β-linked terminal GalNAc, GalNAc α1-3Gal	0.56 ± 0.06	0.51 ± 0.05	1.10	0.78 ± 0.04	0.66 ± 0.02	1.18**
40	Calsepaª	High Man (Man_2-6_), N-glycans including bisecting GlcNAc	0.79 ± 0.05	0.66 ± 0.02	1.21*	0.80 ± 0.05	0.67 ± 0.02	1.20*
41	PTL-I	α-Linked terminal GalNAc	0.48 ± 0.03	0.53 ± 0.05	0.92	0.59 ± 0.04	0.50 ± 0.02	1.17*
42	MAH	Sia α2-3Gal β1-3(Sia α2-6) GalNAc	0.37 ± 0.03	0.49 ± 0.04	0.76*	0.53 ± 0.02	0.50 ± 0.02	1.05
43	WGAª	(GlcNAcβ1-4)_n_, NeuAc, multivalent Sia	1.32 ± 0.09	1.52 ± 0.07	0.87*	1.39 ± 0.02	1.48 ± 0.03	0.93**
44	GSL-IA_4_	α-GalNAc (α-Thr/Ser (Tn))	0.53 ± 0.06	0.43 ± 0.03	1.22	0.70 ± 0.01	0.63 ± 0.02	1.11**
45	GSL-IB_4_ª	α-Gal	0.75 ± 0.02	0.55 ± 0.03	1.35***	0.69 ± 0.01	0.62 ± 0.02	1.11**

^a^Significantly changed in two experiments; * *p* < 0.05; ** *p* < 0.01; *** *p* < 0.001

Among O-glycan binding lectins, signal intensity of ABA, PNA, VVA, SBA, GSL-IA_4_, and GSL-IB_4_ were higher in SP cells, indicating a higher expression of Tn (GalNAc-α-Ser/Thr), T (Galβ1-3 GalNAc-α-Ser/Thr), and sT (sialyl-T) antigens in these cells. Among these lectins, intensities of ABA, VVA, which recognize T, sT and Tn antigens, were the highest in two experiments, respectively. ABA, PNA, VVA, GSL-IB_4_differed significantly in the two experiments. In contrast, SP cells exhibited a significantly lower intensities for (Gal β1-4GlcNAc)_n_ (poly LacNAc) (LEL) and multivalent sialyl glycan binders (WGA).

Among N-glycans, SP cells showed significantly stronger intensity of fucosylation binder AAL, which recognizes the core fucose (Fuc) and Lewis X structure. A higher intensity for AOL, which recognizes both core Fuc and Fuc α1-2 (galactoside β1-4) *n*-acetylglucosamine (Fuc α1-2(Gal β1-4) GlcNAc), was also observed in SP cells. However, SP cells exhibited relatively weak intensity to UEA-I, which recognizes Fuc α1-2(Gal β1-4) GlcNAc. These results together indicated that core fucosylation glycans were increased in SP cells. In addition, the signal for α2-3 linked sialic acids (Sia) binders (MAL) was significantly decreased in SP cells.

### MS analysis of the N-glycan profiles of SP cells and MP cells

**Fig. 3:**
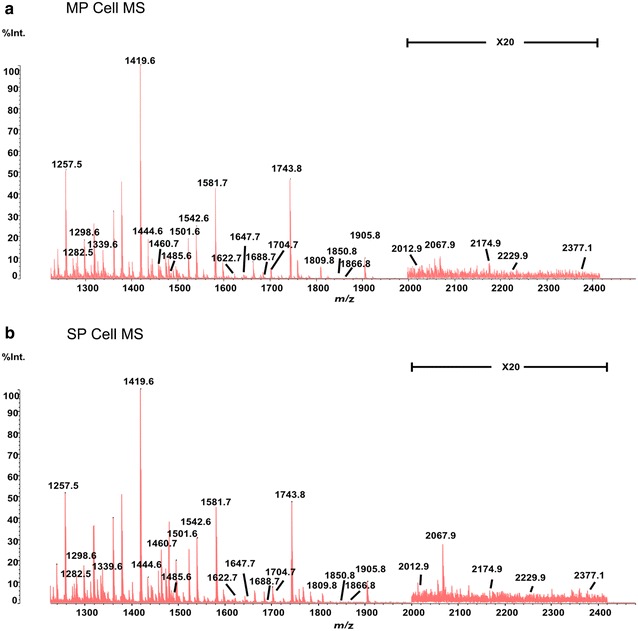
MS spectra of SP cells and MP cells. **a**, **b** The spectra from *m/z* 1250 to *m/z* 2400 of SP cells (*up*) and MP (*down*). Int % of each peak means the relative ion intensity and stand for the ratio of each oligosaccharides’ intensity to the intensity of the highest oligosaccharides

**Fig. 4:**
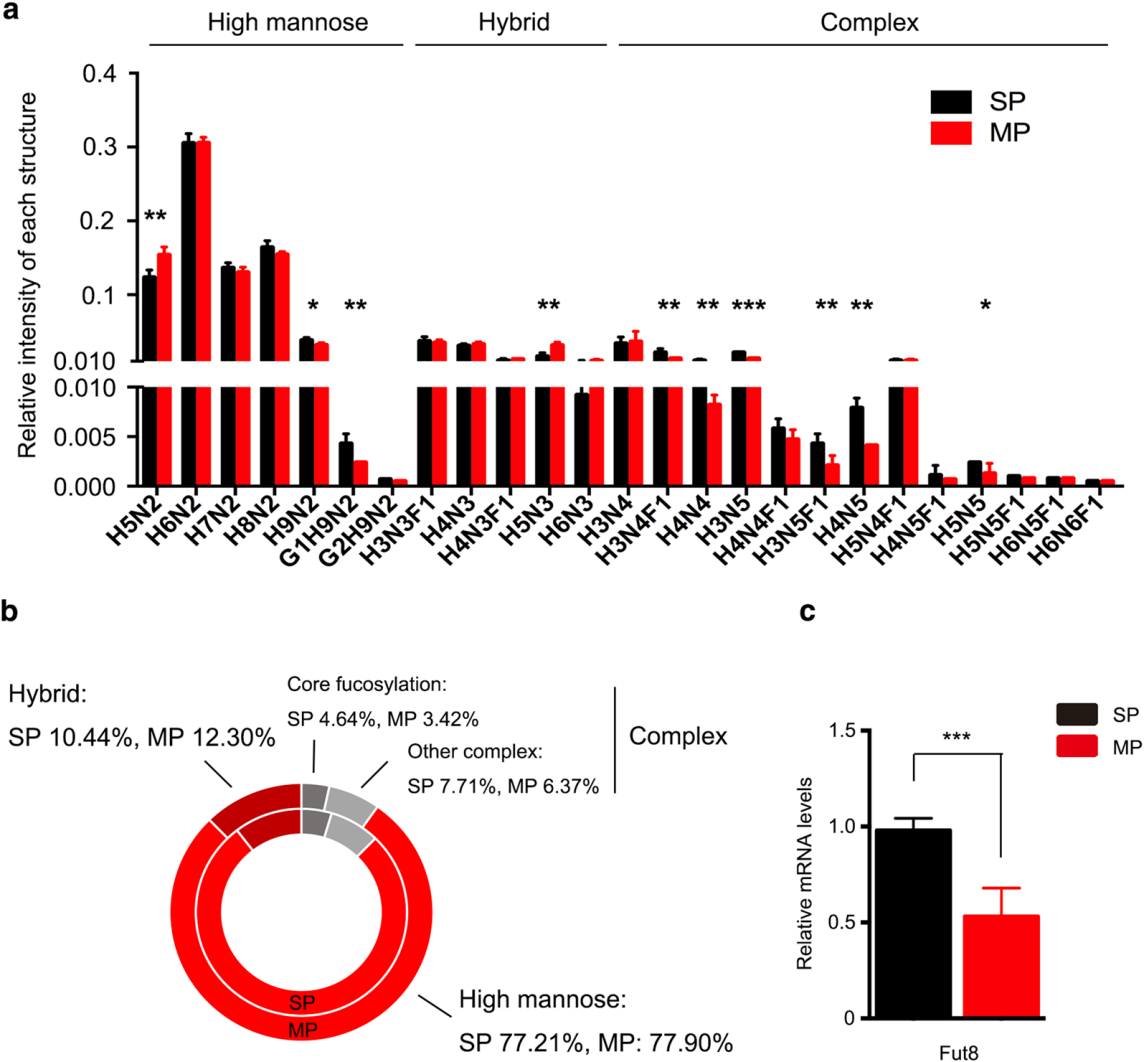
Relative quantities of different classes of N-glycans in SP cells and MP cells. **a** Comparison of the relative quantities of each structure on SP cells and MP cell. The relative quantity of each structure was stand for the proportion of each structure relative intensity in total intensity of all 25 structures. **b** Comparison of the different classes of glycans using a pie chart: the inner circle represents SP cells, and the outer circle represents MP cells. **c** The expression of Fut8 was analysed by quantitative real-time RT-PCR

To obtain more detailed information about the N-glycan structures and their relative intensities, we compared N-glycan profile of the whole proteome of SP cells and MP cells using MS. Twenty-five N-glycans were detected of both cell populations, and were shown in Fig. 3. Statistical analysis was conducted to determine the quantitative differences in glycans between SP and MP cells. A comparison based on different classes (high mannose, hybrid, and complex) were shown in Fig. 4. The relative intensities of each corresponding structure were analyzed by the method above and presented in Table 2.

**Table 2:** Relative intensities of proposed N-glycan structures in SP cells and MP cell

No.	Type	Oberserved *m/z* (M ± Na)	Proposed structure	SP relative intensitymean (n = 5)	RSD (%)	MP relative intensitymean (n = 5)	RSD (%)	Ratio SP/MP	Change (SP/MP)	Composition
1	High mannose	1257.5		0.1235	7.83	0.1538	7.36	0.80**	Down	H5N2
2		1419.6		0.3051	4.16	0.3054	2.63	1.00	Up	H6N2
3		1581.7		0.1362	4.94	0.1304	5.19	1.04	Up	H7N2
4		1743.8		0.1641	5.43	0.1545	2.61	1.06	Up	H8N2
5		1905.8		0.0382	10.92	0.0319	9.08	1.20*	Up	H9N2
6		2067.9		0.0043	26.10	0.0024	12.27	1.74**	Up	G1H9N2
7		2229.9		0.0007	37.67	0.0005	41.18	1.45	Up	G2H9N2
8	Hybrid	1282.5		0.0372	15.05	0.0350	12.52	1.06	Up	H3N3F1
9		1298.6		0.0308	11.24	0.0330	9.10	0.93	Down	H4N3
10		1444.6		0.0108	26.90	0.0127	8.19	0.85	Down	H4N3F1
11		1460.8		0.0165	32.28	0.0316	12.45	0.52**	Down	H5N3
12		1622.7		0.0092	26.98	0.0107	22.17	0.86	Down	H6N3
13	Complex	1339.6		0.0339	26.80	0.0366	37.17	0.93	Down	H3N4
14		1485.6		0.0219	24.14	0.0136	4.33	1.61**	Up	H3N4F1
15		1501.6		0.0110	14.54	0.0082	11.88	1.35*	Up	H4N4
16		1542.6	Or	0.0219	3.77	0.0136	10.59	1.61***	Up	H3N5
17		1647.7		0.0058	21.47	0.0047	20.87	1.22	Up	H4N4F1
18		1688.7	Or	0.0043	21.63	0.0021	39.72	2.04**	Up	H3N5F1
19		1704.7	Or	0.0079	16.73	0.0041	7.03	1.90**	Up	H4N5
20		1809.8		0.0110	14.70	0.0110	20.01	1.01	Up	H5N4F1
21		1850.8	Or	0.0011	56.44	0.0007	27.40	1.49	Up	H4N5F1
22		1866.8		0.0024	20.34	0.0013	47.41	1.91*	Up	H5N5
23		2012.9		0.0010	46.15	0.0008	51.52	1.38	Up	H5N5F1
24		2175.0		0.0008	26.05	0.0008	33.00	1.02	Up	H6N5F1
25		2377.1		0.0005	52.38	0.0005	34.80	0.90	Down	H6N6F1

Values of relative intensity mean represent mean of 5 replicates; symbols for proposed structures: GlcNAc,  , Man,  , Glc,  , Fuc,  , Gal,

*H* hexose, *N N*-acetyl hexosamine, *F* fucose, *G* glucose

We observed that the proportion of high mannose type glycan was nearly identical in SP cells (77.21%) and MP cells (77.90%). Signal of *m/z* 2067.9 was significantly higher (*p* < 0.01, fold change >1.5) in SP cells. In addition, the proportion of hybrid type N-glycan was obviously decreased in SP cells as compared with MP cells (10.44 vs. 12.30%). Most hybrid N-glycans were reduced in SP cells, 1460.8 was significantly decreased (*p* < 0.01, fold change <0.67) in SP cells. However, the percentage of complex N-glycans ranging from *m/z* 1200 to *m/z* 2400 was slightly increased in SP cells (12.35 vs. 9.79%). Among complex N-glycans, core fucosylated N-glycans, catalyzed by fucosyltransferase-8 (FUT8), were also higher in SP cells (4.64 vs. 3.42%), and *m/z* 1485.6 and *m/z* 1688.7 were also significantly higher (*p* < 0.01, fold change >1.5). These results correlated well with the lectin array results (Fig. 2). Moreover, real-time PCR was performed to further verify that SP cells expressed a higher level of FUT8-encoding gene *Fut8* at mRNA level.

### SP cells exhibit higher signal intensity for ABA and VVA

**Fig. 5:**
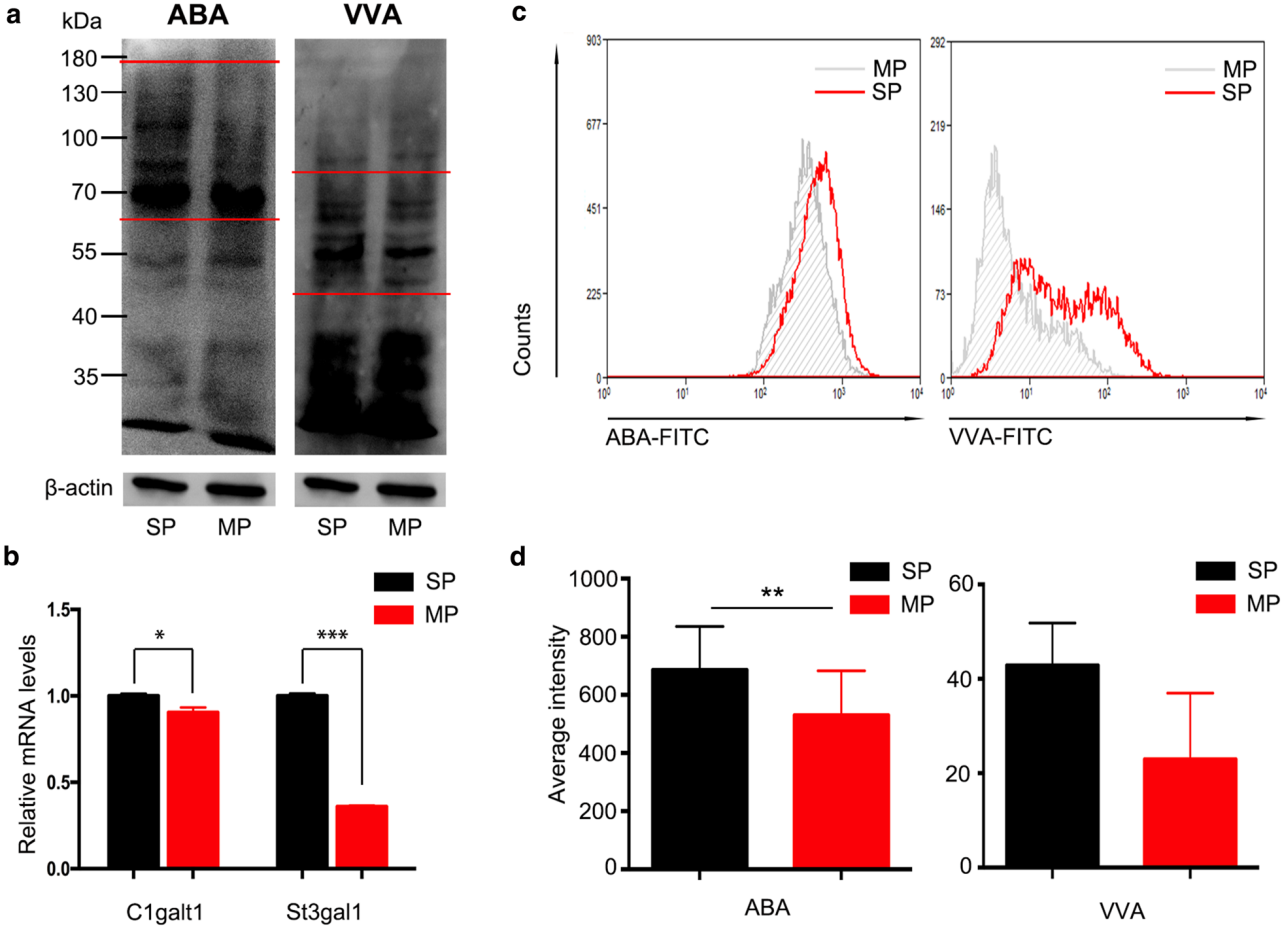
SP cellsexhibited higher intensity of ABA and VVA. **a** Lectin blots were used to detect the expression of the Tn (VVA, *right*), T and ST (ABA, *left*) antigens on SP cells and MP cells, β-actin was performed as control; **b** the mRNA expression levels of C1galt, which acts on Tn to form T antigen, and St3gal1, which forms ST antigen from T, were determined by real-time PCR. **c**, **d** The intensity of ABA and VVA for SP sphere cells after culturing for 5 weeks without serum was detected by flow cytometry and quantified

Lectin array analysis indicated that SP cells expressed more tumor-associated antigens which strongly bound to the lectins ABA and VVA. Among all 45 lectins, ABA, which can recognize both T and sT antigens, exhibited the strongest intensity for SP cells. VVA, which can recognize Tn antigen, also displayed significantly higher intensity for SP cells. Hence, we performed lectin blots to compare recognition of ABA and VVA between SP and MP cells. SP cells exhibited higher intensity for both lectins. For ABA, intensity of glycoprotein from 70–180 kDa was higher in SP cells. For VVA, glycoprotein from 50–80 kDa showed stronger intensity in SP cells (Fig. 5a). These differences were further verified by real-time PCR. C1GALT1 (core 1 synthase, glycoprotein-*N*-acetylgalactosamine 3-beta-galactosyltransferase 1), which acts on Tn to form a T antigen, was increased in SP cells at mRNA level. ST3Gal1 (ST3 beta-galactoside alpha-2,3-sialyltransferase 1), which can modify T antigens to form sT, was also enhanced in SP cells (Fig. 5b).

To investigate whether the SP cells continued to display enhanced binding for ABA and VVA during proliferation, we detected the intensity of ABA and VVA for MP and SP sphere cells after 5 weeks’ culture. Both ABA and VVA continued to strongly bind to SP cells, indicating that these lectins might be potential markers of ovarian cancer SP cells (Fig. 5c, d).

## Discussion

Glycosylation plays important roles in many physiological and pathological processes as well as the identity and function of CSCs. However, the small population size of CSCs has hindered the characterization of glycans released from CSCs. Thus, we used lectin arrays to characterize the entire glycome of CSCs, both N-glycans and O-glycans. Lectin array is time saving (1-day operation), requires small amount of cells (lower limit of 1000 cells). And it can analyze intact glycoproteins, which simplified sample preparation and made detection of sialic acid easy. We also used MS to characterize the structure of N-glycans and provide information about the composition of the N-glycome. Combination of these two methods provided more comprehensive information about glycans in ovarian CSCs. In this study, we didn’t conduct MS analysis of O-glycans, because it needs 10^6^–10^7^ cells at least for analysis [24], and it was difficult to enrich enough HO8910 pm SP cells. New methods or technologies are still needed to overcome these obstacles.

Our present study suggested that SP cells may overexpress the tumor-associated antigens, T, Tn, and sT. The lectin arrays revealed enhanced intensity of VVA, SBA, and GSL-IA lectins in SP cells, which implied Tn antigen overexpession in SP cells. Increased expression of C1GALT1 and signal intensity of ABA and PNA lectins in SP cells suggested upregulation of T antigen. Besides, T antigens were modified by ST3Gal1, which was enhanced in SP cells, to form sT antigen recognized by ABA lectin. Our lectin blot results also confirmed stronger intensity of ABA, VVA for SP cells. Furthermore, after 5 weeks’ culture without serum, the SP sphere cells still displayed higher intensity of ABA and VVA compared with MP cells, indicating that the expression of these antigens continued during CSC proliferation. To further verify whether these changes were only HO8910 pm cell line specific, we used another ovarian cancer cell line ES-2 for SP cell sorting and validated that ES-2 SP cells also showed higher intensity of ABA and VVA than ES-2 MP cells (see Additional file 1).

In our MS analysis, glycans with terminal Glcs (glucose), which are usually related to glycoprotein quality control [25, 26] and exhibit higher expression in cancer [27, 28], were expressed more abundantly in SP cells. Increased hybrid N-glycans were detected in MP cells, indicating that the immediate products of GlcNAc transferase I were not further modified by other GlcNAc transferases. Among more mature N-glycans, core fucosylation N-glycans were more abundant in SP cells, as verified by MS and lectin array. The mRNA transcript level of FUT8, which synthesises the core fucosylation, was also enhanced. Increased core fucose is closely associated with many types of cancer. It showed an unfavourable clinical outcome and played as a functional regulator of non-small cell lung cancer [29, 30]. Loss of FUT8 also down-regulates several cellular signaling pathways to inhibit chemical-induced hepatocellular carcinoma [31].

The common available biomarkers of ovarian CSCs are ALDH, CD133+, CD24+ CD44+, CD44+ CD117+ and negative Hoechst 33342 stain. The use of a single marker has disadvantages because not all cancer cell lines or tissues are positive for the marker and the cells enriched for a marker maybe not purified. In addition, ALDH, CD133, CD44, and CD177 are also expressed in normal stem cells. Thus, new biomarkers are still needed, and glycans may provide a breakthrough.

This study implied higher levels of Tn, T and sT antigens in ovarian cancer SP cells. Tn, T and sT antigens are weakly expressed in normal tissues but overexpressed in most human carcinomas and are associated with poor prognosis [32–34]. A recent review by Karsten and Goletz [19] hypothesized that T antigen might be a potential biomarker of CSCs. Because this antigen is only expressed in onco-foetal stem cells and tumor cells, targeting T antigen should not harm normal adult stem cells. Lin et al. also confirmed the co-expression of T antigen with the stem cell markers CD44 and CD133 in lung, breast and liver cancer [35].

Our results are similar to those of previous lectin studies of CSCs. In glioblastoma, lectins recognizing GalNAc and GlcNAc exhibit higher affinity for the surface of undifferentiated cells, suggesting that GalNAc and GlcNAc might be a novel biomarker to identify CSCs [19]. In the same study, the lectins PNA, GSL-I, SBA, and VVA also exhibited higher signals in CSCs. In pancreatic CSCs, Terao et al. observed enhanced ABA and VVA intensity of CSCs using the same lectin array [36]. In addition, liver CSCs isolated as CD133^+^CD13^+^cells also exhibited stronger intensities of SNA, SSA, ABA, PNA, and SBA [18].

Based on the results of the present study and previous reports, we hypothesize that tumor-associated glycan antigens may be novel potential CSC markers. Future studies should examine whether lectin ABA combined with available markers can isolate more malignant CSCs.

## Conclusions

In conclusion, we conducted a systematic glycomic analysis of ovarian cancer SP cells using lectin arrays and MS. Our findings suggested that SP cells expressed increased core fucose, Tn, T and sT antigens. In addition, hybrid type, α2,3-linked sialic glycan, and multivalent sialyl-glycan were enhanced in MP cells. Aberrant glycan profiles may be related to some stemness properties of SP cells and may provide potential biomarkers for ovarian cancer CSCs (all lectins in the array: see Additional file 2).

## Additional files

**Additional file 1.** SP cells sorted from ES-2 cells exhibited higher intensity of ABA and VVA. The intensity of ABA (left) and VVA (right) for SP sphere cells after culturing for 1weeks without serum was detected by flow cytometry.

**Additional file 2.** The category of representative lectins for glycan profiling.

## Abbreviations

CSCs: cancer stem cells
SP: side population
MP: main population
PI: propidium iodide
MS: mass spectrometry
Fuc: fucose
Gal: galactose
GalNAc: *N*-acetylgalactosamine
Glc: glucose
ACN: acetonitrile
TFA: trifluoroacetic acid
PBS: phosphate-buffered saline
SDS-PAGE: sodium dodecyl sulfate-polyacrylamide gel electrophoresis
PVDF: polyvinylidene difluoride
ABA: *Agaricusbisporus*
VVA: *Viciavillosa*
PCR: polymerase chain reaction
SSA: *Sambucussieboldiana*
FUT8: fucosyltransferase-8
C1GALT1: core1synthase, glycoprotein-*N*-acetylgalactosamine 3-beta-galactosyltransferase1
ST3Gal1: ST3 beta-galactoside alpha-2,3-sialyltransferase 1
Tn antigen: GalNAc α-Ser/Thr
T antigen: Galβ1-3GalNAc α-Ser/Thr (Thomsen–Friedenreich antigen)
sT antigen: sia α2-3Gal β1-3GalNAc α-Ser/Thr (sialyl-T)
