# Supplementary material for: Integrated glycomic analysis of ovarian cancer side population cells
Source: Clin Proteomics. 2016 Nov 10;13:32. doi: 10.1186/s12014-016-9131-z (PMC5103398; doi:10.1186/s12014-016-9131-z)
Supplement: Supplementary file 1 — Additional file 1. SP cells sorted from ES-2 cells exhibited higher intensity of ABA and VVA. The intensity of ABA (left) and VVA (right) for SP sphere cells after culturing for 1weeks without serum was detected by flow cytometry. [file 12014_2016_9131_MOESM1_ESM.docx]

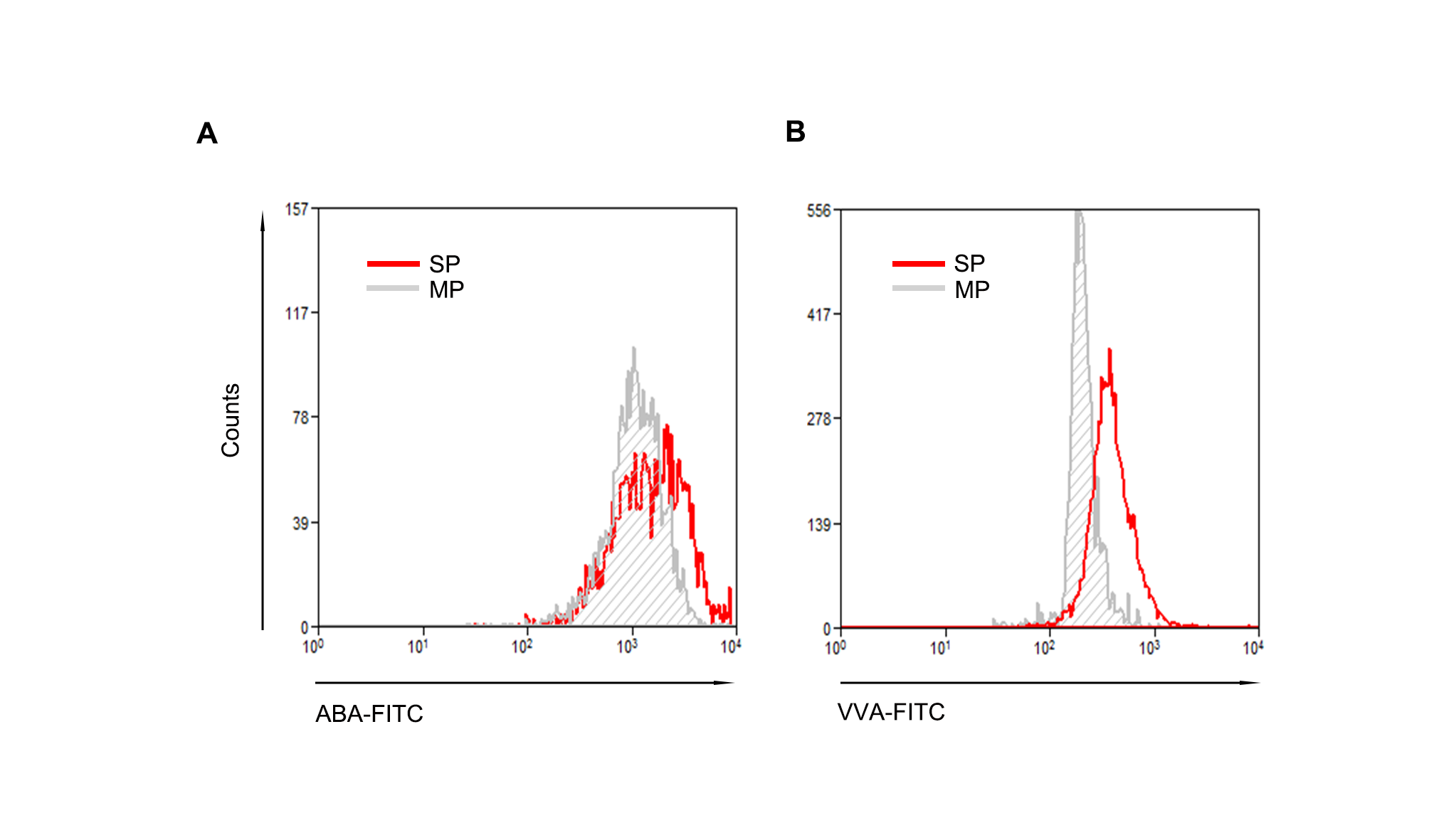
Additional file 1. **SP cells sorted from ES-2 cells exhibited higher intensity of ABA and VVA**

The intensity of ABA (left) and VVA (right) for SP sphere cells after culturing for 1weeks without serum was detected by flow cytometry.
